# A figure of merit for transcranial ultrasound transmission based on coupled elastic-thermal analysis

**DOI:** 10.1088/1361-6560/ae1ee3

**Published:** 2025-11-24

**Authors:** M Sait Kilinc, Costas Arvanitis, F Levent Degertekin

**Affiliations:** 1 School of Electrical and Computer Engineering, Georgia Institute of Technology, Atlanta, GA 30332, United States of America; 2 G.W. Woodruff School of Mechanical Engineering, Georgia Institute of Technology, Atlanta, GA, United States of America; 3 Wallace H. Coulter Department of Biomedical Engineering, Georgia Institute of Technology and Emory University, Atlanta, GA 30322, United States of America

**Keywords:** transcranial focused ultrasound, mode conversion, lamb waves, transmission, skull absorption, skull heating

## Abstract

*Objective.* To evaluate the efficiency of ultrasound transmission and its associated thermal effects on the skull, introducing a figure of merit (FOM) to assess the efficacy and safety of transcranial focused ultrasound (tFUS) at varying incidence angles and frequencies. *Approach*. A coupled elastic-thermal analysis was performed using a two-dimensional (2D) parallel solid plate skull model, validated through three-dimensional simulations. High-resolution micro-computed tomography (microCT) data from an *ex vivo* human skull sample was incorporated for realistic modeling. Simulations were conducted at frequencies of 0.8 MHz, 1 MHz, and 1.2 MHz to explore the impact of skull thickness, incidence angle, and absorption properties on ultrasound transmission and heating. The FOM was calculated for each scenario to provide an integrated measure of transmission efficiency and thermal safety. Experimental studies using the same *ex vivo* human skull validated the microCT-based simulation model. *Main results*. The FOM analysis demonstrated that at 0.8 MHz for an 8 mm thick skull, the FOM dropped by half for incidence angles above 15°, indicating reduced ultrasound transmission and heightened temperature rise at oblique angles. These findings suggest that dual-mode conversion at higher angles may not be suitable for tFUS applications, supporting the clinical preference for near-normal incidence. The validated 2D microCT model provides a reliable framework for studying the interplay between transmission and heating effects. *Significance*. This study provides an integrated framework for optimizing tFUS parameters by balancing transmission efficiency and thermal safety. The introduced FOM offers a novel metric to guide therapeutic applications, contributing to safer and more effective transcranial ultrasound treatments.

## 1. Introduction

Transcranial-focused ultrasound (tFUS) is a non-invasive technique for diagnostic and therapeutic applications in the brain. More specifically, the use of tFUS for therapy is an emerging field of research accelerated with recent US Food and Drug Administration approvals of commercial systems (Elias *et al* 2016). This method has been used for treatment of essential tremor and tremor-dominant Parkinson’s Disease (Bond *et al* 2017, Martinez-Fernandez *et al* 2020), and it has been investigated for thermal ablation of tumors (McDannold *et al* 2010), and drug delivery in the brain through temporary opening of the blood-brain barrier (BBB) in tumors (McMahon *et al* 2019, Anastasiadis *et al* 2021) and neurological disorders (Lipsman *et al* 2018, Rezai *et al* 2020). In addition to drug delivery, tFUS-BBB opening when combined with liquid biopsy techniques holds promise for minimally invasive diagnosis of brain diseases (Meng *et al* 2021, Yuan *et al* 2023).

The central premise of all these applications is safely delivering sufficient ultrasound energy through the skull in a spatially and temporally controlled fashion. This is complicated by the large variability of skull bone geometry and properties and the complex viscoelastic interaction between ultrasound waves and the skull (Hynynen and Clement 2007). Particularly, absorption of ultrasound energy in the skull bone leads to heating and local temperature rise which can lead to overheating of the skull scalp and regions of the brain next to the skull bone (Connor and Hynynen 2004, Pernot *et al* 2004, Schwartz *et al* 2018, McDannold *et al* 2020). To avoid excessive heating, a hemisphere-shaped phased array transducer is employed to distribute the acoustic energy over the skull area (Clement *et al* 2000). The array elements are densely distributed and driven with different amplitudes and phases to compensate for wavefront aberrations caused by the skull layer, enabling precise focusing while minimizing heating (Connor and Hynynen 2004). This array geometry ensures that the ultrasound waves are incident to the skull bone at an angle close to the normal. When the skull geometry relative to certain array elements prohibits normal incidence, those elements are turned off (McDannold *et al* 2010). With this approach, however, the targetable region is limited to deep, central locations in the brain. Depending on the anatomical target, some patients may not be eligible for the therapy because many array elements need to be inactivated, causing suboptimal sonication (Chang *et al* 2016). In addition to the incidence angle, other factors that diminish ultrasonic energy transmission include a skull density ratio of less than 0.40 (Chang *et al* 2016, Boutet *et al* 2019), a skull volume greater than 330 cm^3^ (Lipsman *et al* 2013, Chang *et al* 2016, Schwartz *et al* 2018, Wang *et al* 2018, Boutet *et al* 2019), and hyperostosis (Chang *et al* 2016, Boutet *et al* 2019, Bernstock *et al* 2022). Three-dimensional (3D) printed helmet scaffolds have been proposed to drive only longitudinal waves as the system conformally cover the skull and enable near-normal incidence of multiple phased array modules (Adams *et al* 2021), but these are still at the proof of concept stage and their ability to target brain regions close to the skull surface needs to be determined.

By realizing that the skull bone behaves like a plate, oblique incidence transmission through dual mode (longitudinal to shear or Lamb wave to longitudinal mode) conversion methods have been proposed to improve ultrasound transmission (Kang *et al* 2022, Mazzotti *et al* 2023). The main premise of the plate mode conversion approach is the excitation of so-called Lamb waves when the incidence angle of the longitudinal waves matches the critical angle of a particular Lamb wave mode (Bartoli *et al* 2006) (also see appendix A for a simplified model calculation). Several recent studies have investigated the dual mode conversion approach under various assumptions, suggesting that using ultrasound waves obliquely incident on the skull can improve transmission and better target peripheral regions of the brain for treatment. In one case, oblique incidence is used to promote shear mode ultrasound propagation in the skull bone (Clement *et al* 2004, White *et al* 2006) with the aim of maintaining the coherency of the transmitted beam due to a low impedance mismatch between shear waves in the skull and longitudinal waves in the brain, which could theoretically lead to a better spatial resolution and, in certain cases, a higher transmission. While at certain frequencies (300 kHz) transmission improvements at specific incidence angles in the parietal region of the human skull can be attained (Kang *et al* 2022), the findings to date seem to be highly dependent on the particular skull sample and local geometry (Kang *et al* 2022, Mazzotti *et al* 2023). Moreover, when a high porosity is assumed in a layered skull model, significant transmission loss is observed with oblique incidence (Jing and Lindsey 2021).

These studies, however, primarily use pressure transmission as the main metric of comparison and do not address the critical issue of local temperature elevation in the skull. Local temperature elevation during transcranial ultrasound procedures mainly in thermal ablation trials remains a significant concern (Connor and Hynynen 2004, Odeen *et al* 2014, Krishna *et al* 2018, Schwartz *et al* 2018, McDannold *et al* 2020) and the primary motivator for such approaches, as it limits the amount of acoustic power that can be safely delivered to the targeted brain region (Odeen *et al* 2014, Hughes *et al* 2016, Boutet *et al* 2018). Therefore, a more comprehensive analysis of the thermal impact of incidence angle and mode conversion is needed to optimize acoustic power transfer while considering the maximum allowable temperature rise in the skull.

In this study, we investigate ultrasound transmission through skull sample and its associated thermal impact using both simulations and experimental studies with *ex vivo* human skull sample, leading to the development of a figure of merit (FOM) that integrates these two phenomena. We begin by performing numerical acoustic simulations using coupled elastic/thermal models on a parallel solid plate structure, considering different skull thicknesses (6 mm, 8 mm, and 10 mm), frequencies (0.8 MHz, 1 MHz, and 1.2 MHz), and a range of absorption values for longitudinal and shear waves based on the literature (Connor and Hynynen 2004, White *et al* 2006, Pinton *et al* 2012, Attali *et al* 2023). This allows us to capture possible transmission and thermal effects. Next, we incorporate high-resolution micro-computed tomography (microCT) data of an *ex vivo* human skull for a more realistic representation of the skull structure, which is then validated through experimental studies within the same frequency range. Finally, we introduce the FOM using the microCT-based model. By conducting a comprehensive analysis of the combined effects of ultrasound transmission and temperature rise on transcranial ultrasound at varying angles of incidence, the findings from this study are expected to offer valuable guidance for patient selection and optimization of transcranial ultrasound therapy.

## 2. Methods

### 2.1. Analysis of ultrasound transmission and thermal effects in skull layers using elastic and thermal simulations

#### 2.1.1. Simulation framework for elastic and thermal analysis of ultrasound transmission through parallel solid plate skull models

**Figure 1.**
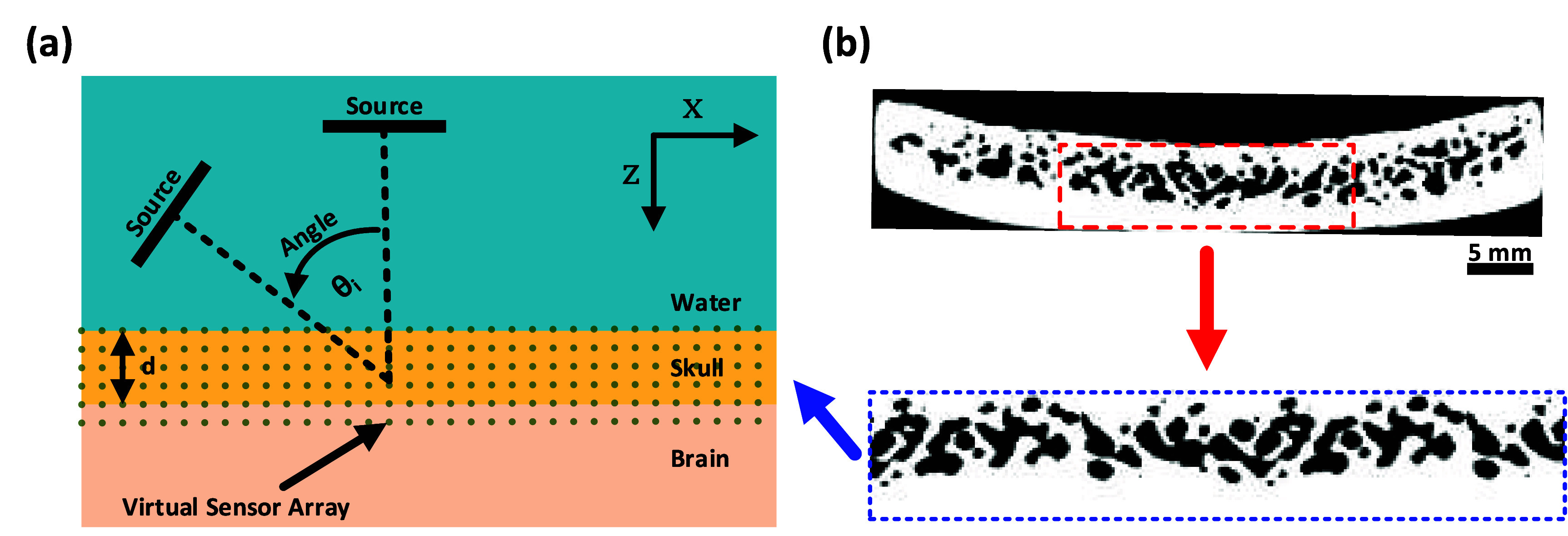
(a) Schematic of the 2D simulation model used for elastic and thermal simulations. (b) Extraction of a representative 2D cross-sectional region from the microCT scan of the skull. The highlighted section (red dashed box) from the full skull cross-section (top) is isolated and transformed into a parallel microCT-based skull model (blue dashed box, bottom) for place in the 2D simulation setup in skull layer, accurately preserving the microstructural details for ultrasound transmission and heating analysis.

In this study, elastic and thermal simulations were conducted using the Kelvin-Voigt elastic wave model and the bio-heat equation via k-Wave simulation tool (Treeby *et al* 2014). The objective was to investigate the transmission of finite ultrasound beams through a water/skull/brain structure as a function of the angle of incidence while mapping the temperature rise in the skull to establish a FOM. A comparative analysis was conducted using high-resolution two-dimensional (2D) simulations (0.05 mm grid size) and low-resolution 2D and 3D simulations (0.5 mm grid size) based on a solid parallel plate model figure 1 to evaluate the impact of spatial resolution and simulation dimensionality on the accuracy of the results.

The parallel solid plate models of the skull were simulated for three commonly reported thicknesses: 6 mm, 8 mm, and 10 mm. Although most simulations utilized a single homogeneous layer to represent the skull while considering semi-infinite water and brain tissue layers, this simplified model has been deemed effective for tFUS simulations (Jones and Hynynen 2016). Additionally, microCT-based simulations were conducted for further validation and are detailed in section 2.1.5. The acoustic and thermal parameters used in these simulations were sourced from the literature (Fry and Barger 1978, Kinsler *et al* 2000, Clement *et al* 2004, White *et al* 2006, Treeby and Cox 2014) and are provided in table 1. Our experimental results, supported by literature (White *et al* 2006, Treeby and Cox 2014), indicate that assuming a higher shear wave absorption is more realistic. Therefore, in this study, the shear wave absorption coefficient was set to nearly twice that of the longitudinal wave, but results for the equal absorption case are included in the Appendix to cover range of different absorption ratios. Since the acoustic properties of water and brain tissue exhibit highly similar characteristics with negligible absorption loss (Goss *et al* 1978, Duck 1990b, Clement *et al* 2004), their acoustic medium properties were considered identical (Fry and Barger 1978, Duck 1990a). However, in thermal simulations, they were modeled differently due to more significant differences in thermal properties. Additionally, all acoustic and thermal properties were assumed to be temperature-independent throughout the simulations. While this assumption is commonly applied in transcranial ultrasound modeling, it may not be valid for high-energy therapeutic applications where temperature-dependent changes in material properties become significant (Hughes *et al* 2018).

**Table 1.** Elastic and thermal material parameters used in the simulations.

Parameter	Degassed water	Skull bone	Brain tissue	Citation
Longitudinal wave speed [m s^−1^]	1481	2850	1481	Fry and Barger (1978), Kinsler *et al* (2000), White *et al* (2006)
Shear wave speed [m s^−1^]	N/A	1400	N/A	White *et al* (2006)
Longitudinal wave absorption [dB (MHz^2^ cm)^−1^]	0	8.83 ^a^, 8.83 ^b^	0	Clement *et al* (2004), White *et al* (2006), Treeby and Cox (2014)
Shear wave absorption [dB (MHz^2^ cm)^−1^]	N/A	19.5 ^a^, 8.83 ^b^	N/A	White *et al* (2006), Treeby and Cox (2014)
Density [kg m^−3^]	998	1732	998	Fry and Barger (1978), Kinsler *et al* (2000)
Thermal conductivity [W (m.K)^−1^]	0.628	0.43	0.51	Fry and Barger (1978), Duck (1990a)
Specific heat [J (kg.K)^−1^]	4182	1440	3640	Fry and Barger (1978), Duck (1990a)

^a^ Shear dominant absorption coefficient condition.

^b^ Identical absorption coefficients condition.

The diagram of the 2D simulation model is illustrated in figure 1(a). The ultrasound source was modeled as a single-element transducer with a 12.7 mm element size, matching the dimensions used in our experiments. The source positioned at an adjustable angle (${\theta _i}$) relative to the skull surface, with the water-skull-brain layers indicated. The distance between the transducer and the skull was maintained at 30 mm to allow for variation in the incidence angle from 0° (normal incidence) to 60°, with a 2° step resolution. A continuous sinusoidal wave with a constant surface acoustic intensity of 5 W cm^−2^, corresponding to an incident free-field pressure of approximately 500 kPa at the skull surface, was transmitted at center frequencies of 0.8 MHz, 1 MHz, and 1.2 MHz, each with a Gaussian envelope. These frequencies are slightly higher than typical tFUS systems, were selected to emphasize the effects of mode conversion, as a greater number of Lamb waves are generated at higher frequencies. Additionally, the higher frequencies enable tighter focusing, which can be important for targeting brain structures located near the skull. A virtual sensor array is positioned within the skull and brain regions to record the various parameters. The variable d represents the thickness of the skull layer, and the *X*–*Z* coordinates define the simulation axes. For the 3D simulations, the model illustrated in figure 1(a) was extended along the $y$ -axis, with the source geometry adjusted to match the circular transducer used in the experiments.

The 2D simulation domains had matrix sizes of 2048 × 1024 (for 0.05 mm grid size) and 256 × 128 (for 0.5 mm grid size), while the 3D model used a matrix size of 128 × 256 × 128 with a 0.5 mm grid spacing. A runtime of 80 *µ*s was employed to achieve a steady-state condition, determined based on the simulation domain’s dimensions and verified through a convergence study. A perfectly matched layer was implemented with 20 points around each edge of the domain to absorb any outgoing waves. Given the high absorption rates and the necessity for numerical stability, a Courant–Friedrichs–Lewy (CFL) condition of 0.055 was selected, ensuring that the simulation adhered to stability constraints without excessive computational cost (Treeby and Cox 2014).

A convergence study was conducted to determine appropriate grid resolution, time step, and runtime, ensuring that further refinements would not significantly alter the results. The study evaluated different spatial resolutions (0.5 mm, 0.1 mm, 0.05 mm grid sizes), CFL values (0.1, 0.05, 0.025), and simulation durations (60 *µ*s, 80 *µ*s, 100 *µ*s). The results demonstrated that 80 *µ*s was sufficient to capture steady-state conditions without unnecessary computational overhead, while 0.05 mm spatial resolution and CFL = 0.055 provided numerically stable and accurate results.

Given the extensive number of simulations required, the computational workload was distributed and executed in parallel using a computer cluster equipped with 64GB of RAM and a single Intel Xeon E3-1245 v6 quad-core CPU running at 3.7 GHz. Separate simulations were conducted for each 2° incidence angle, transmit frequency, skull model, and absorption case to systematically evaluate their effects on ultrasound transmission and heating. In total, over 1300 individual simulations were performed. The simulations were executed in MATLAB, with computation times varying based on grid size, dimensionality, and absorption inclusion. For 2D simulations with a 0.5 mm grid size, the average runtime was approximately 1 min per simulation, while for a finer 0.05 mm grid size, it increased to 3 h. In 3D simulations with a 0.5 mm grid size, the computation time extended to about 1.5 h per simulation. To efficiently manage the computational demands, simulations were optimized for parallel execution.

#### 2.1.2. Simulation framework with high-resolution microCT model

To further validate our findings and provide a more realistic assessment of ultrasound transmission and heating in the skull, we conducted simulations using high-resolution microCT data of *ex vivo* human skull sample used in the experiments. Prior to scanning, the skull sample was degassed in a vacuum chamber overnight to eliminate air bubbles within its porous structure. The microCT scans were performed using a Skyscan 1173 system with a voxel resolution of 0.048 mm, enabling accurate modeling of the complex porous geometry of the skull. The sample used was approximately 8 mm thick and obtained from an *ex vivo* human skull (Skulls Unlimited International Inc., Oklahoma City, OK, USA). It belonged to a 60 year-old male individual with an unknown medical history and was stored in an aqueous formaldehyde solution (10% buffered formalin) prior to the microCT scan. No identifying information was collected, and institutional review board approval was not required.

Due to the computational burden of 3D simulations at micrometer resolution, a 2D cross-section of the skull was extracted from the microCT dataset. A rectangular region of the microCT image was inserted into the 2D model, with matrix sizes of 2048 × 1024 and grid spacing of 0.05 mm. The microCT image was segmented to preserve the pores of the trabecular bone. The extracted image and its transfer into a plate model are illustrated in figure 1(b). The pores were modeled as being filled with degassed water, with longitudinal wave speed and density matching those of water. The material properties used in the simulations were sourced from table 1. The rest of the simulation setup was the same as described in section 2.1.1.

#### 2.1.3. Calculation of acoustic power transmission coefficients

To calculate the acoustic power transmission, the time-varying components of the acoustic intensity vector were recorded via a virtual receiver array that was placed in the brain region both in the presence and absence of skull layers. Average acoustic intensity values were extracted from the recorded data and integrated along a line in 2D simulations and over a plane in 3D simulations. These integrated intensity values were then divided to calculate the power transmission coefficients for each corresponding frequency, incidence angle, and skull thickness as

**Figure 2.**
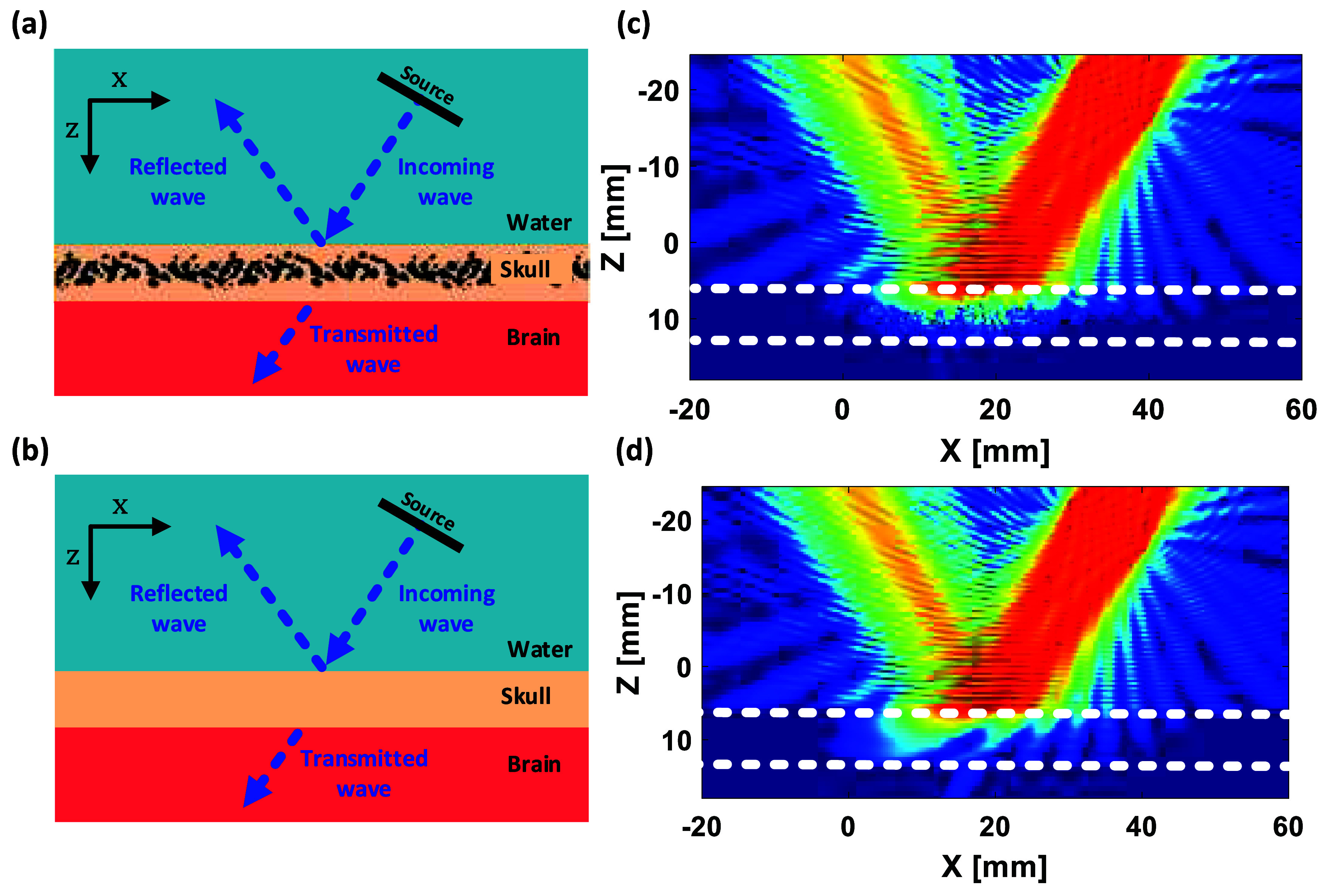
Schematic representation and normalized maximum pressure field comparisons between the parallel solid plate skull model and the microCT-based skull model. (a) and (b) Illustrate the conceptual diagrams of ultrasound wave interactions with the skull, including reflection, absorption, and transmission. (c) and (d) Show the corresponding simulated pressure maps for the parallel solid plate skull model and the microCT-based skull model, respectively. The pressure fields are normalized to their maximum values, with the skull layer indicated by white dashed lines. The results highlight the differences in wave propagation, including transmission loss and scattering effects, between the two skull models.

\begin{equation*}T\left( {f,{\theta _{\text{i}}},d} \right) = \frac{{{{\mathop \sum \nolimits}}{{}_x}{I_{\text{S}}}\left( {x;f,{\theta _{\text{i}}},d} \right)}}{{{{\mathop \sum \nolimits}}{{}_x}{I_{\text{i}}}\left( {x;f,{\theta _{\text{i}}},d} \right)}}\end{equation*}

where ${I_{\text{S}}}\left( {x;f,{\theta _i},d} \right)$ indicates the average acoustic intensity measurements in the brain at the skull/brain interface in the presence of the skull layer, ${I_{\text{i}}}\left( {x;f,{\theta _{\text{i}}},d} \right)$ is the free-field (without the skull) average acoustic intensity measurements, is the axis in the 2D simulation (replaced with in the 3D simulations), $f$ is the center frequency of the input signal, ${\theta _{\text{i}}}$ is the angle of incidence, and $d$ is the thickness of the skull layer. Note that intensity ${I_{\text{S}}}$ is calculated through particle velocity and pressure fields, which includes all scattering due to internal structure (porosity) and reflection from the skull surface due to the contrast between the skull bone and medium properties, in addition to the absorption in the skull bone structure. The reflection and scattering effect can be seen in figure 2.

#### 2.1.4. Calculation of temperature rise in skull layers

To calculate the temperature rise in the skull layer, a virtual receiver array was inserted into the skull layer to capture the time-varying particle velocity in all directions. For each simulation, the volume rate of heat deposition was calculated from the particle velocity and other medium properties according to the plane wave relationship, thereby coupling the elastic simulations with the thermal simulations. This relationship is defined as

\begin{equation*}{Q_{{\text{ext}}}} = \alpha I = \alpha \rho c{u^2}\end{equation*}

where ${Q_{{\text{ext}}}}$ is external volume rate of heat deposition, $\alpha $ is absorption coefficient, $\rho $ is mass density, $c$ is speed of sound, and $u$ is steady state particle velocity amplitude, which is extracted from the time-varying particle velocity. This particle velocity field includes all scattering due to internal structure (porosity) and reflection from the skull surface due contrast between the skull bone and medium properties.

The generation of heat in the skull results from the absorption of the waves in the skull, including multiple scattering. *k*-Wave enables analysis of the impact of each wave type on heating by shear and longitudinal waves separately, through separate calculations of the external heat source term in the bio-heat equation. This was performed by splitting the steady-state shear and longitudinal particle velocity amplitudes in each elastic simulation. The overall external heat source can then be calculated by considering these individual contributions as

\begin{equation*}{Q_{{\text{ext}}}} = {Q_{\text{L}}} + { }{Q_{\text{S}}}\end{equation*}

where ${Q_{\text{L}}}$ and ${Q_{\text{S}}}$ are contributions of longitudinal and shear waves on heat deposition, respectively. Once the elastic simulations reached a steady state, the instantaneous ${Q_{{\text{ext}}}}$ was calculated over three cycles of recordings.

Subsequently, the thermal simulations using the same spatial matrix as in the elastic simulations were executed for a duration of 10 s, with a step size of 10 ms which represents a typical treatment time (McDannold *et al* 2019). The 10 ms time step was determined through a convergence study to ensure a balance between accuracy and computational efficiency, confirming that further reductions in step size did not significantly alter the temperature distribution while increasing computational cost. The temperature distribution throughout the skull layer was modeled using Pennes’ bio-heat transfer equation (Pennes 1948) as described

\begin{equation*}\rho C\frac{{\partial T}}{{\partial t}} = .\left( {\kappa T} \right) + {Q_{{\text{ext}}}}\end{equation*}

where $C$ is the specific heat capacity of the medium, $T$ is the temperature, $t$ is time, $\kappa $ is thermal conductivity. A uniform background temperature of 37 °C was assigned prior to sonication, and the heat dissipation in the water and brain was considered in the solution without considering perfusion. Similarly, the effect of circulating cooling water is not considered. Although this impacts the absolute accuracy of the temperature rise, the focus here is on the ratio of temperature rise at different incidence angles.

#### 2.1.5. FOM analysis

To comprehensively evaluate the performance of ultrasound transmission through the skull, we introduce FOM that combines two critical factors: ultrasound power transmission and maximum temperature rise in the skull layers. The former quantifies the proportion of ultrasound power that successfully transmits through the skull and reaches the brain tissue, whereas the latter serves as an indicator for assessing the safety of ultrasound procedures. The overall goal is to achieve high ultrasound transmission while minimizing temperature rise for a given input power level. Thus, a natural FOM emerges from the ratio of these two parameters, providing a figure that enables straightforward comparison. Additionally, this ratio is multiplied by the integrated average acoustic intensity to account for the overall power delivery as a function of frequency ($f$), incidence angle (${\theta _{\text{i}}}$), skull thickness ($d$). Therefore, FOM is defined as

\begin{equation*}{\text{FOM}}\left( {f,{\theta _{\text{i}}},d} \right) = \left| {\mathop {\sum\limits}\limits_x {I_{{\text{SS}}}}\left( {x;f,{\theta _{\text{i}}},d} \right)} \right|\frac{{T\left( {f,{\theta _{\text{i}}},d} \right)}}{{{\text{max}}\left( {H\left( {x,z;f,{\theta _{\text{i}}},d} \right)} \right)}}\quad\left( {{\text{W}}/\left( {{\text{m}}^\circ {\text{C}}} \right)} \right)\end{equation*}

where $H\left( {x,z;f,{\theta _{\text{i}}},d} \right)$ is peak temperature rise map in 2D simulations ($H\left( {x,{ }y,z;f,{\theta _{\text{i}}},d} \right){ }$ in 3D simulations) and ${I_{{\text{SS}}}}\left( {x;f,{\theta _{\text{i}}},d} \right)$ is the integrated average acoustic intensity incident on skull layer’s outer surface in 2D simulations (${I_{{\text{SS}}}}\left( {x,y;f,{\theta _{\text{i}}},d} \right)$ in 3D simulations). This metric assesses both the efficacy and safety of transcranial ultrasound procedures, offering a valuable tool for optimizing therapeutic ultrasound applications.

### 2.2. Experimental validation of ultrasound transmission through *ex vivo* human skull sample

**Figure 3.**
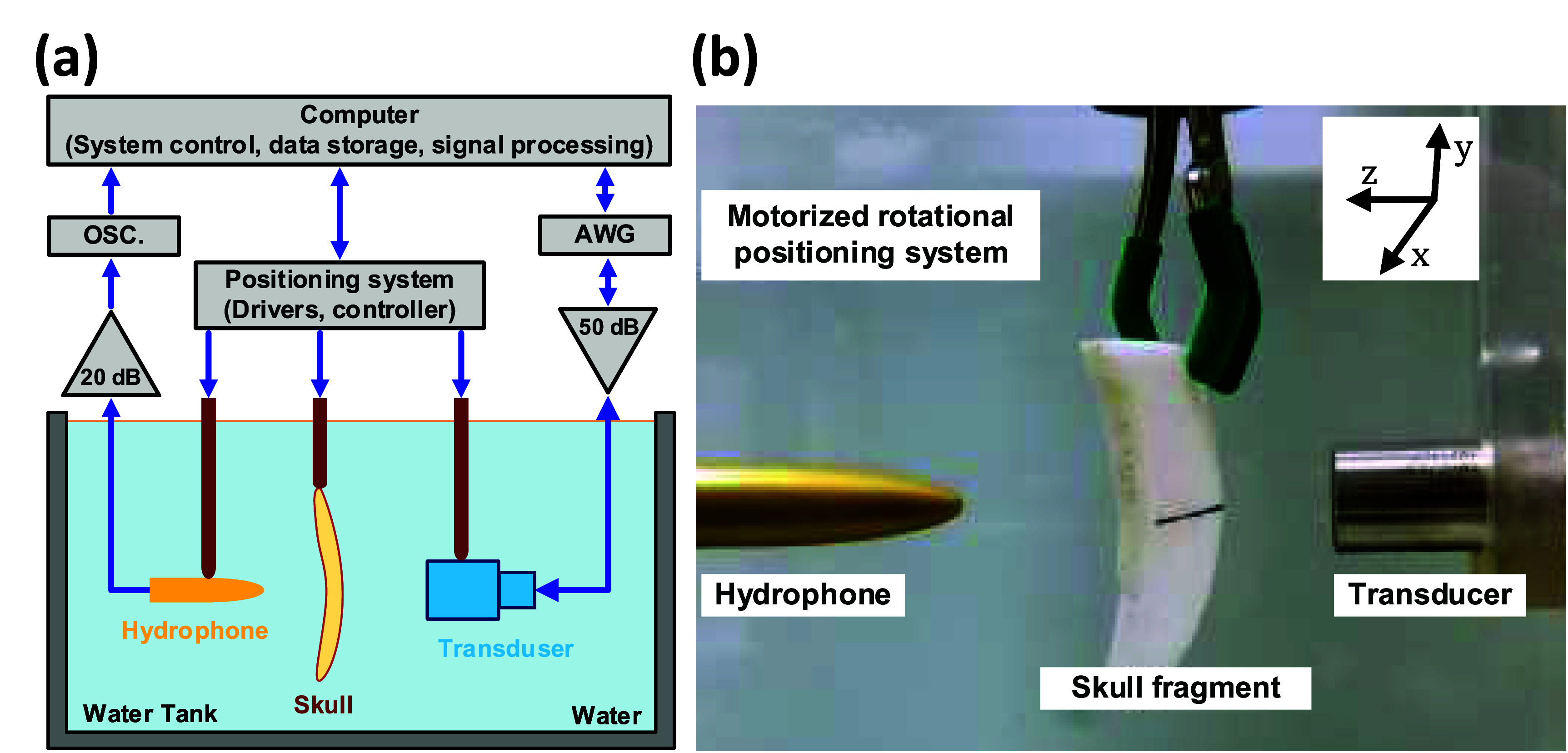
Experimental setup for measuring ultrasound transmission through *ex vivo* skull sample. (a) The schematic diagram on the left illustrates the experimental configuration. (b) The photograph on the right shows the physical arrangement of the setup with the skull fragment placed between the transducer and the hydrophone. The motorized rotational positioning system enables precise adjustment of the transducer’s angle of incidence for the ultrasound transmission measurements.

To validate our simulation results, we conducted a series of ultrasound transmission experiments using *ex vivo* human skull sample. The objective was to estimate which absorption set was more realistic to simulate and compare these experimental results with our simulation data. A series of ultrasound transmission experiments were conducted within a water tank environment as shown in figure 3. The sample for these experiments was obtained from an *ex vivo* human calvaria, specifically from the parietal region, with a bone segment measuring approximately 5 × 20 cm. This segment was cleaved to facilitate measurements at high incidence angles (∼55°) within the experimental setup. The same skull sample was used to generate the microCT dataset. To ensure the absence of bubbles within the porous structure of the skull, the sample was placed in degassed water in a vacuum chamber overnight prior to the experiments. The experiments were conducted in degassed, room-temperature water, with the water tank walls positioned far from the specimen to minimize the occurrence of standing waves.

The sample was mounted between a broadband plane-wave transmitting transducer of 1 MHz, 60% fractional bandwidth, and circular 12.7 mm diameter aperture (Olympus, Shinjuku City, Tokyo, Japan, model A303S) and a 0.085 mm diameter aperture polyvinylidene fluoride hydrophone (ONDA, Hammerwood Avenue, Sunnyvale, California, USA model HGL-0085). The transducer surface was positioned approximately 26 mm from the outer surface of the skull fragment, the distance measured with pulse-echo measurements, which prevents the formation of standing waves between the transducer and the skull surface. The hydrophone was situated 110 mm from the transducer surface which avoids hydrophone-to-skull sample collision at high incidence angles.

An arbitrary waveform generator (Keysight, Santa Clara, California, USA, model 33220A), amplified by 50 dB with a power amplifier (Electronic Navigation Industries, New York, USA, model 310l) was used to drive the transducer. A burst sine wave (0.8 MHz, 1.0 MHz, 1.2 MHz, 50 cycles, pulse repetition rate 200 Hz) was generated and transmitted through each of the skull specimen. The acoustic field was measured with a hydrophone, and the waveform as registered by the hydrophone was amplified by 20 dB (ONDA, Hammerwood Avenue, Sunnyvale, California, USA model AG-2010), connected to a digital oscilloscope (Keysight, Santa Rosa, California USA, model DSOX1102 G), and transferred to a computer (Dell, Round Rock, Texas, USA) for further analysis. To mitigate interference effects caused by the long pulse duration, a Hamming window was applied to the signals during post-processing, ensuring accurate extraction of transmission coefficients. The window was applied to the time region where the signal was expected to arrive, as the coordinates of both the hydrophone and the transducer were known, allowing precise isolation of the relevant signal components.

The transmission experiments were conducted using a custom positioning system capable of performing rotational scanning with precise step sizes. The system and other measurement devices were synchronized to facilitate automated measurements. The scan dimensions were chosen based on preliminary 1D scans to ensure complete coverage of the transmitted beam profile across all incidence angles, capturing pressure levels down to −24 dB of the transmission coefficients. Since this criterion was consistently applied to both experiments and simulations, further increasing the scan dimensions would not impact the results but would only extend the experiment duration, as transmission remains unchanged beyond this threshold. The skull sample was mounted on a rotary arm, allowing for precise control of the incidence angle of the impinging ultrasound wave from the transmitting transducer. The rotary arm could scan an angle of incidence ranging from −45.9° to +45.9°, with a step size of 2.7° (precision ±0.45°). The study was conducted at three different excitation frequencies of 0.8 MHz, 1 MHz, and 1.2 MHz. Two different mounting procedures were followed during the intracranial ultrasound field measurements. First, the transducer and hydrophone were positioned without the skull sample, and the hydrophone location was aligned to the maximum pressure output in a plane perpendicular to the direction of the beam. Second, the skull segment was positioned between the transducer and hydrophone using pulse-echo measurements. The maximum echo signal was assumed to correspond to the location of the skull surface perpendicular to the transducer, and the orientation of the skull was adjusted accordingly. However, due to natural skull curvature, local deviations from normal incidence may still exist. This limitation has been acknowledged as a potential source of variation in experimental measurements. For each combination of angle of incidence, 2D scan measurements were performed both in the free field and in the presence of the skull sample at different frequency outputs. The experimental ultrasonic transmittance was calculated by dividing the total power values over the measurement plane for each of the three excitation frequencies.

Unlike most previous studies that relied on single-point measurements, the ultrasound transmission coefficients in this study were calculated for each excitation frequency and incidence angle using the results obtained from a 2D field distribution with and without the skull sample. After collecting the data, the transmission coefficients were determined based on the integration of average intensity values across the scanned field. The acoustic intensity was integrated over the x and y coordinates using the harmonic plane wave assumption, and calculated as follows

\begin{equation*}{T_{{\text{average}}}}\left( {f,{\theta _{\text{i}}}} \right) = \frac{{\mathop {\sum\limits}\limits_x \mathop {\sum\limits}\limits_y \left( {\frac{1}{t}\mathop \smallint \limits_0^t {\raise0.7ex\hbox{${{p_{\text{s}}}{{\left( {x,y;f,{\theta _{\text{i}}}} \right)}^2}}$} \!\mathord{\left/ {\vphantom {{{p_{\text{s}}}{{\left( {x,y;f,{\theta _{\text{i}}}} \right)}^2}} {2{\rho _{\text{w}}}{c_{\text{w}}}}}}\right.} \!\lower0.7ex\hbox{${2{\rho _{\text{w}}}{c_{\text{w}}}}$}}} \right)}}{{\mathop {\sum\limits}\limits_x \mathop {\sum\limits}\limits_y \left( {\frac{1}{t}\mathop \smallint \limits_0^t {\raise0.7ex\hbox{${{p_o}{{\left( {x,y;f,{\theta _{\text{i}}}} \right)}^2}}$} \!\mathord{\left/ {\vphantom {{{p_o}{{\left( {x,y;f,{\theta _{\text{i}}}} \right)}^2}} {2{\rho _{\text{w}}}{c_{\text{w}}}}}}\right.} \!\lower0.7ex\hbox{${2{\rho _{\text{w}}}{c_{\text{w}}}}$}}} \right)}}\end{equation*}

where $t$ is time duration, ${p_{\text{s}}}\left( {x,y;f,{\theta _{\text{i}}}} \right)$ is the pressure field measurements in the water in presence of the skull layer, ${p_0}\left( {x,y;f,{\theta _{\text{i}}}} \right)$ is the free field (without the skull) pressure field measurements, $\left( {x,y} \right)$ are the coordinates in the water medium, $f$ is the center frequency of input signal, ${\theta _{\text{i}}}$ is the angle of incidence, ${\rho _{\text{w}}}$ is density of water, and ${c_{\text{w}}}$ is speed of sound in water.

## 3. Results

In this section, we begin by comparing the acoustic power transmission coefficients obtained from both 2D and 3D parallel solid plate model simulations. These results are compared across various incidence angles and frequencies to evaluate the accuracy and reliability of using 2D simulations as a simplified representation of the more computationally intensive 3D models. Next, we validate the microCT model by comparing its acoustic power transmission coefficients with experimental results at different incidence angles and frequencies. Following this, we present thermal simulations using the microCT model to demonstrate how temperature distribution changes as a function of incidence angle and frequency. Finally, we introduce the FOM results, a combined metric that evaluates both transmission efficiency and thermal effects within the microCT model.

### 3.1. Validation of ultrasound transmission model

**Figure 4.**
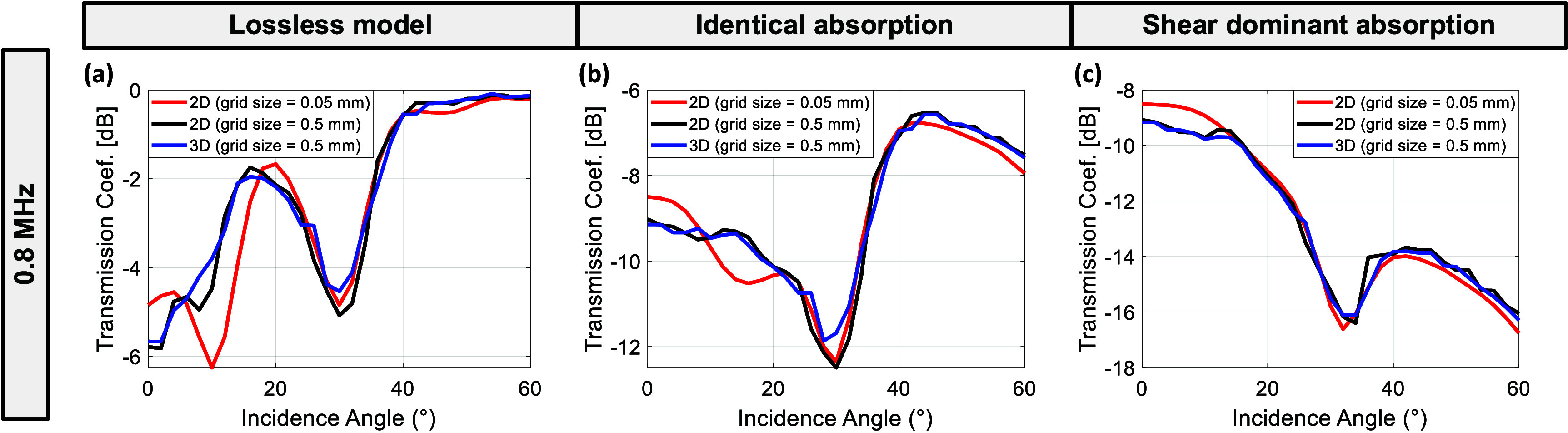
Comparison of transmission coefficients for 2D and 3D simulations with different grid sizes for an 8 mm skull thickness at 800 kHz. (a) Lossless model, (b) identical absorption coefficients for longitudinal and shear waves, (c) shear-dominant absorption coefficients. The 2D simulations with a 0.05 mm grid size (red) are compared to the 2D simulations with a 0.5 mm grid size (black) and 3D simulations with a 0.5 mm grid size (blue).

The transmission coefficient results of the 2D and 3D simulations with a 0.5 mm grid size compared to those with a 0.05 mm grid size for an 8 mm parallel solid plate skull model 800 kHz are shown in figure 4. In figure 4(a), representing the lossless model, all three scenarios show similar trends, with transmission peaks and dips at comparable incidence angles. The 2D simulation with a 0.05 mm grid size (red) shows minor deviations at low incidence angles, while the 2D and 3D simulations with a 0.5 mm grid size (black and blue) are closely aligned. This indicates that the 2D simulations with coarser grid sizes can reasonably capture the transmission behavior in the absence of absorption losses. Figure 4(b), corresponding to the identical absorption model, shows a more pronounced effect of absorption on transmission coefficients. The trends remain similar for all three scenarios, but there is a noticeable reduction in transmission compared to the lossless model. The alignment between 2D and 3D models at higher incidence angles suggests that even with identical absorption, the 2D models with 0.5 mm grid spacing can approximate the transmission characteristics of 3D models. In figure 4(c), where the shear wave absorption dominates, the transmission coefficient decreases significantly at higher incidence angles. The 2D and 3D models show good agreement in this scenario, with minor discrepancies around 10°–20° incidence angles. This suggests that for shear-dominant scenarios, 2D simulations provide a reliable approximation of the 3D model, for the grid sizes considered here.

**Figure 5.**
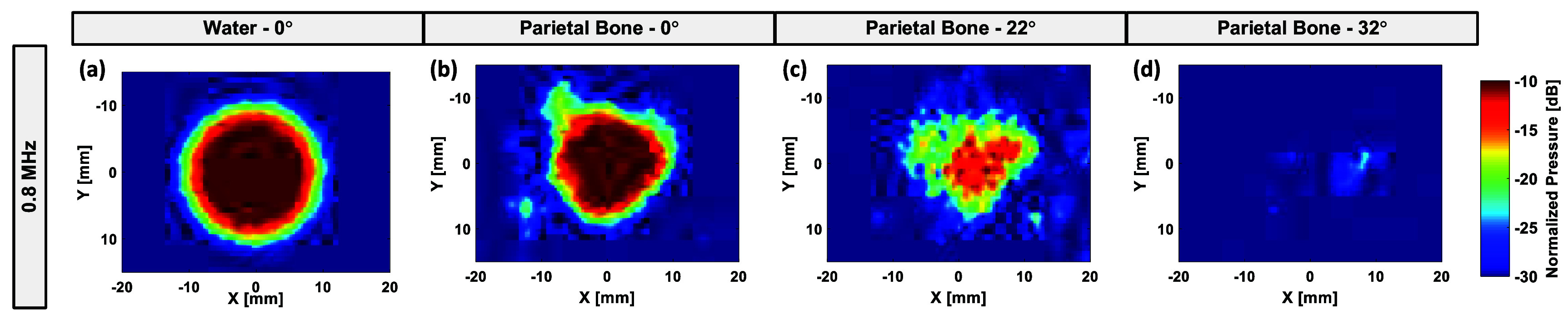
Pressure field distributions at 0.8 MHz for varying conditions. (a) Pressure field in water without the skull at 0° incidence angle, demonstrating a symmetric beam pattern. (b) Pressure field through the parietal bone at 0° incidence angle, showing slight beam distortion. (c) Pressure field through the parietal bone at 22° incidence angle, displaying increased beam distortion. (d) Pressure field through the parietal bone at 32° incidence angle, illustrating severe beam distortion. The figures are normalized and the pressure scale (dB) indicates the transmission loss at different angle.

Figure 5 illustrates the measured pressure field distributions at 0.8 MHz under different conditions. In figure 5(a), the pressure field in water without the skull at 0° incidence angle shows a well-defined, symmetric beam pattern with the highest intensity at the center. This serves as the reference condition for evaluating the impact of the skull on ultrasound propagation. When the ultrasound beam encounters the skull at 0° incidence, as shown in figure 5(b), the pressure field becomes less uniform, with noticeable beam distortion and reduced intensity compared to the water-only condition. This reduction is primarily due to the reflection and scattering caused by the skull’s heterogeneous structure. As the incidence angle increases to 22° (figure 5(c)), the beam experiences further distortion and reduction in intensity. The pressure field shows more complex scattering patterns and a broader distribution of energy, indicating significant disruption in beam coherency due to oblique transmission through the skull. At 32° incidence angle (figure 5(d)), the pressure field displays severe beam distortion and further reduction of intensity. The increased scattering and reduced pressure magnitude at higher incidence angles indicate that the skull’s impact on ultrasound propagation becomes more pronounced, resulting in a highly diffused and fragmented pressure field distribution. These findings were used to derive experimental transmission coefficients, which were subsequently compared to the microCT-based transmission results to further validate our simulation models.

**Figure 6.**
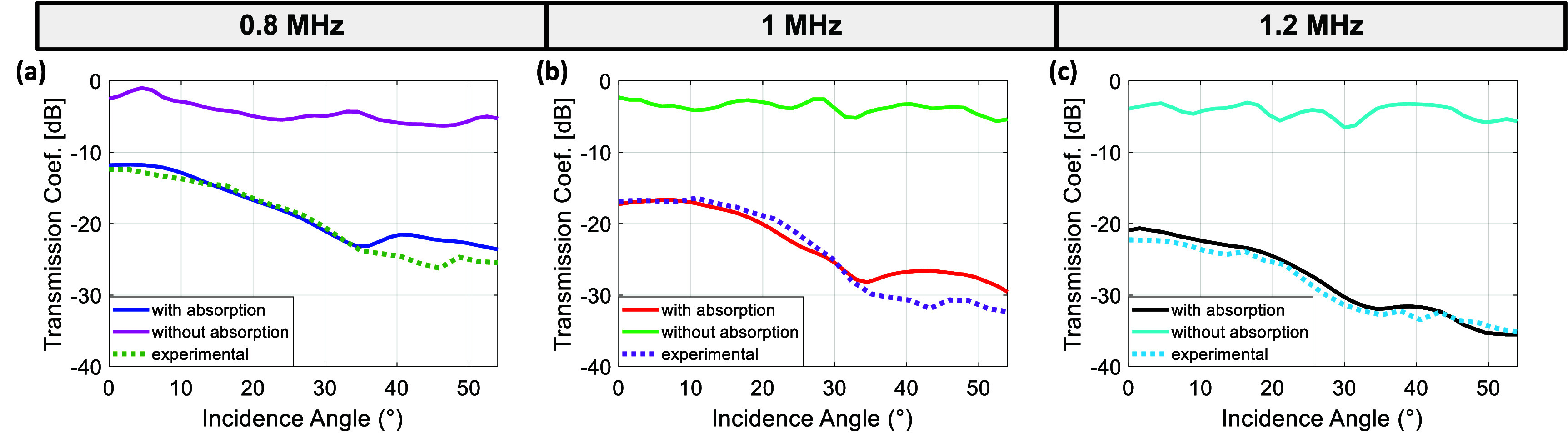
Transmission coefficients as a function of incidence angle for simulations with shear wave dominant absorption in microCT model, without absorption in microCT model, and experimental measurements at three different frequencies: (a) 0.8 MHz, (b) 1 MHz, and (c) 1.2 MHz.

The comparison of acoustic power transmission coefficients are given in figure 6, which presents results for simulations conducted with and without absorption, alongside the corresponding experimental measurements.

At 0.8 MHz (figure 6(a)) and 1 MHz (figure 6(b)), the transmission coefficient values obtained from the experimental measurements generally align with those of the simulations incorporating absorption across most incidence angles. However, deviations are observed around 40°–45°, where the experimental results differ from the simulations with absorption by approximately 4 dB at 0.8 MHz and 5 dB at 1 MHz. Despite this discrepancy, the inclusion of absorption in simulations improves agreement with the experimental data, highlighting the significant role of absorption in ultrasound transmission loss through the skull. At 1.2 MHz (figure 6(c)), the simulation results with absorption closely match the experimental data across all incidence angles, with a maximum deviation of only 2 dB near 40°. In contrast, the simulation results without absorption consistently overestimate the transmission coefficient across all incidence angles, reinforcing the necessity of incorporating absorption effects for realistic modeling. At 0.8 MHz (figure 6(a)), the transmission coefficient values obtained from the experimental measurements are closely aligned with those of the simulations that include absorption across all incidence angles, showing a consistent trend. This alignment suggests that the absorption properties are accurately captured in the model. The simulation results without absorption show significantly higher transmission coefficients, indicating that absorption in the skull plays a major role in attenuating ultrasound transmission. Similarly, at 1 MHz (figure 6(b)), the experimental transmission coefficients show good agreement with the simulations incorporating absorption. The results further confirm that absorption is the primary contributor to ultrasound attenuation. Without considering absorption, the simulation values deviate significantly from both the experimental and absorption-included simulation results. At 1.2 MHz (figure 6(c)), the general trend is maintained, with simulations including absorption closely matching the experimental results, while the simulations without absorption overestimate the transmission coefficient across all incidence angles.

### 3.2. Thermal simulations of transcranial ultrasound transmission

**Figure 7.**
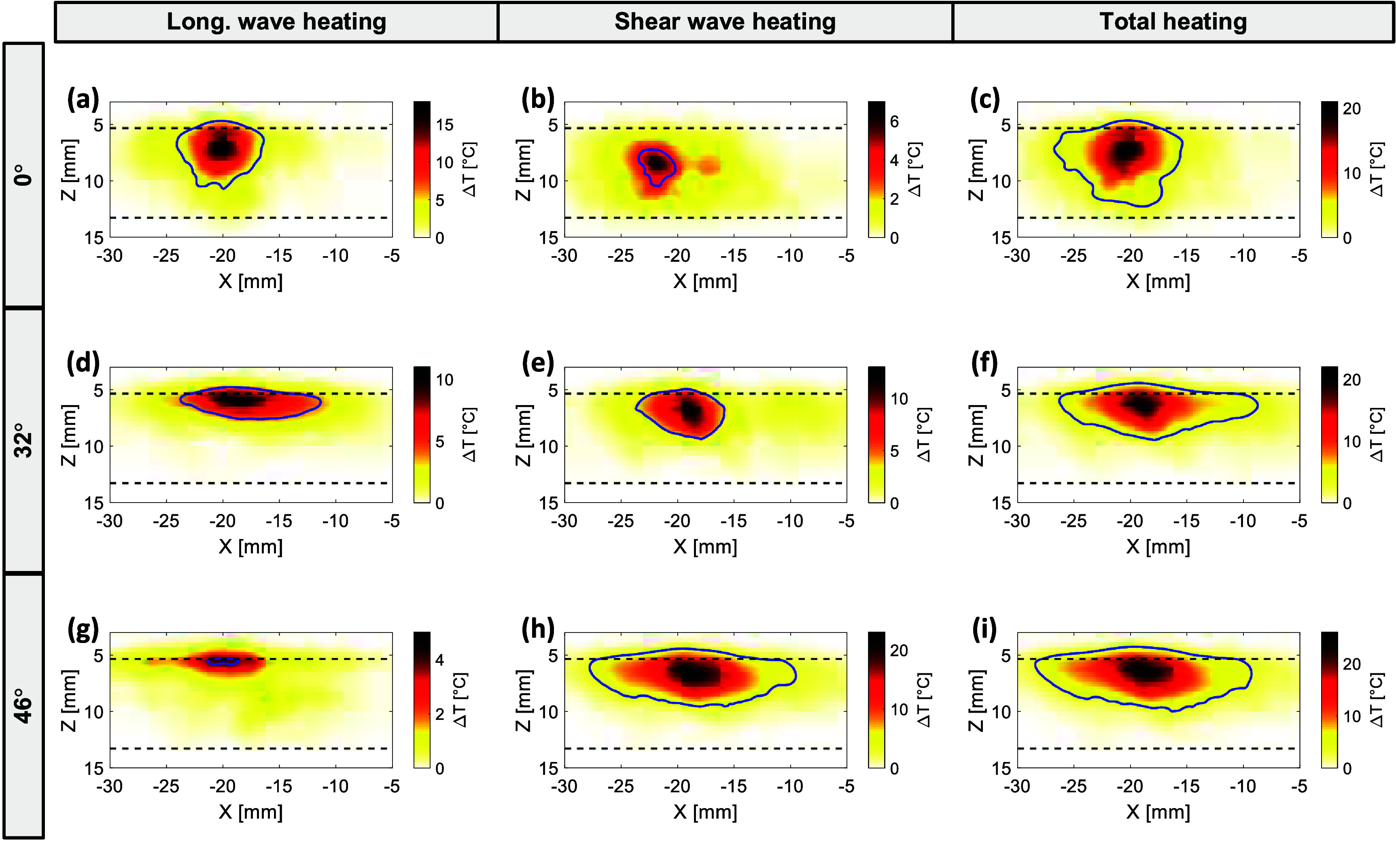
Temperature rise distributions in the skull segment for different incidence angles (0°, 32°, and 46°) at 0.8 MHz, highlighting contributions from longitudinal wave heating (left column), shear wave heating (middle column), and total heating (right column) using the shear wave-dominant absorption model in the microCT-based simulation. The blue contours indicate regions with a temperature rise of 4 °C.

As the microCT model with shear wave-dominant absorption was validated in the previous section for acoustic transmission, thermal simulations were subsequently performed using this model while acknowledging that the absorption parameters were assumed rather than directly validated through experimental temperature measurements. Figure 7 illustrates the temperature rise distributions in the microCT segment due to heating from longitudinal and shear waves at three different incidence angles (0°, 32°, and 46°). The left column (figures 7(a), (d) and (g)) represents the heating contributions from longitudinal waves, the middle column (figures 7(b), (e) and (h)) shows the heating contributions from shear waves, and the right column (figures 7(c), (f), and 6(i)) presents the total temperature rise, which is a combination of both wave types.

At normal incidence (0°), the temperature rise is primarily governed by the longitudinal wave heating, as seen in figure 7(a), with a peak temperature increase of approximately 18 °C. The contribution from shear wave heating in figure 7(b) is minimal, resulting in a total temperature rise of around 21 °C, as shown in figure 7(c). This indicates that at normal incidence, shear wave conversion is minimal, and the temperature rise is dominated by longitudinal wave components. When the incidence angle increases to 32° (figures 7(d)–(f)), the temperature distribution becomes more uniform along the lateral direction, and the contribution of shear wave heating increases significantly, reaching a maximum of 13 °C in figure 7(e). The total temperature rise shown in figure 7(f) is around 22 °C, with more evident lateral heat spreading compared to normal incidence. At a 46° incidence angle (figures 7(g)–(i)), the shear wave heating becomes even more prominent, reaching a peak temperature rise of 13 °C in figure 7(h), contributing significantly to the total temperature rise of 26 °C in figure 7(i). The results indicate that at oblique incidence angles, the shear wave heating component plays a larger role in the overall temperature distribution. The temperature rise is more widespread along the skull surface, suggesting that increased shear wave conversion leads to higher and more distributed heating at these angles.

**Figure 8.**
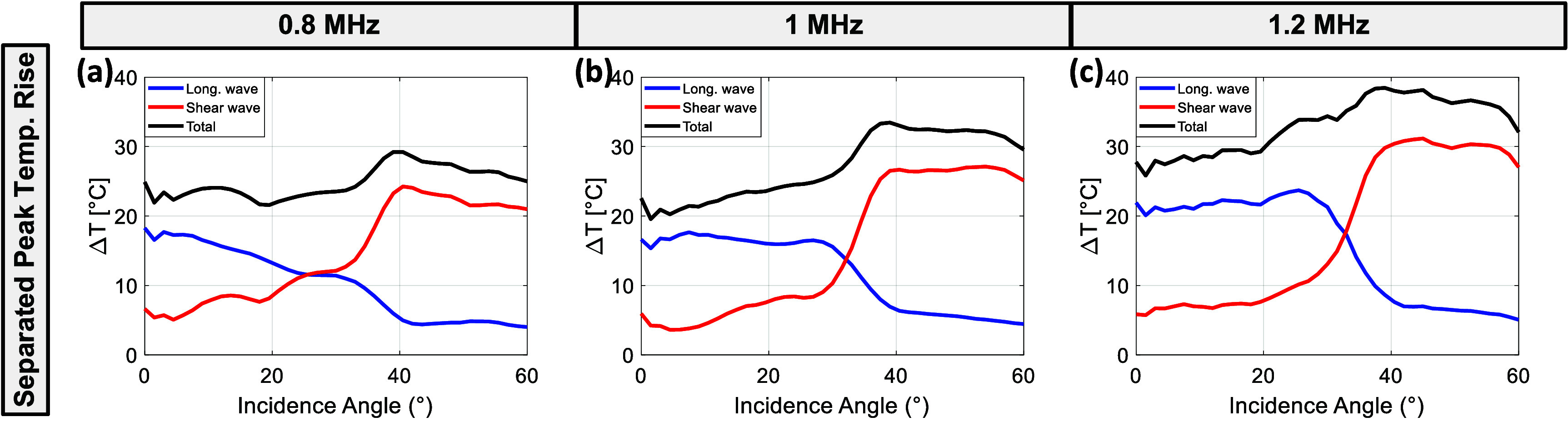
Separated peak temperature rise as a function of incidence angle for longitudinal wave heating (blue), shear wave heating (red), and combined total heating (black) at three different frequencies in shear wave dominant absorption in microCT model: (a) 0.8 MHz, (b) 1 MHz, and (c) 1.2 MHz.

Based on the above results in figures 7 and 8 presents the separated peak temperature rise values in the skull at different incidence angles for 0.8 MHz, 1 MHz, and 1.2 MHz, obtained using the microCT-based thermal simulations. The contributions of longitudinal wave heating (blue), shear wave heating (red), and total heating (black) are displayed as a function of incidence angle for each frequency.

At 0.8 MHz (figure 8(a)), the longitudinal wave heating decreases at higher angles. In contrast, the shear wave heating component remains relatively low at smaller angles but starts to increase sharply beyond 20°, peaking at approximately 40° incidence and contributing more to the overall heating than the longitudinal waves at higher angles. This results in a maximum total temperature rise of around 30 °C at an incidence angle of 40°. For 1 MHz (figure 8(b)), a similar trend is observed, where the longitudinal wave heating rises slightly at lower angles, peaking around 10° and then gradually decreasing with increasing angle. The shear wave heating shows a more rapid increase with incidence angle, surpassing the longitudinal component beyond 30°. The total temperature rise peaks at around 35 °C at approximately 39° incidence angle, demonstrating the increasing contribution of shear waves at higher angles. At 1.2 MHz (figure 8(c)), the longitudinal wave heating remains approximately same with increasing incidence angle until 26°, becoming negligible beyond 30°. In contrast, the shear wave heating dominates, leading to a sharp increase in total temperature rise up to 40° incidence. The total peak temperature rise reaches its maximum of approximately 38 °C at around 38°, with a slight decline observed at larger angles.

**Figure B1.**
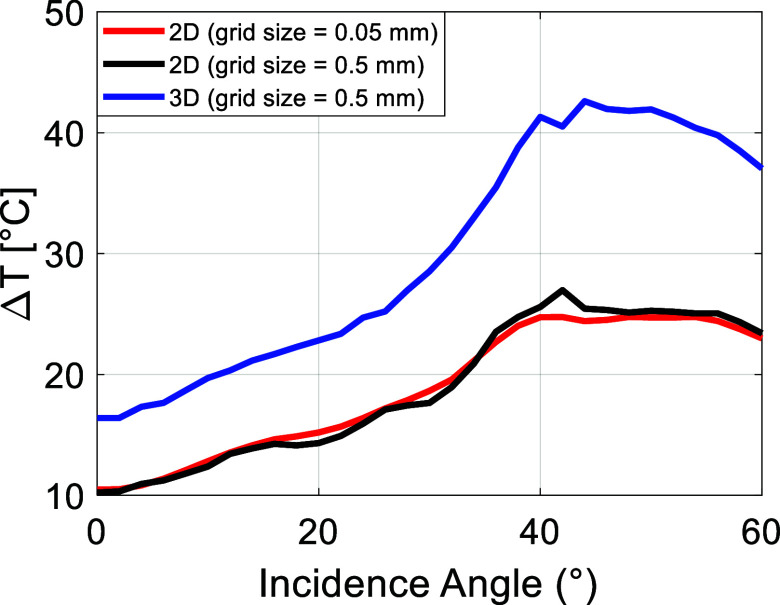
Total temperature rise as a function of incidence angle for 2D and 3D simulations of parallel solid plate skull models subjected to 0.8 MHz ultrasound, using shear-dominant absorption coefficients. Results are presented for 2D simulations with a 0.05 mm grid size (red), 2D simulations with a 0.5 mm grid size (black), and 3D simulations with a 0.5 mm grid size (blue).

The impact of spatial resolution and dimensionality on temperature rise was further analyzed, as detailed in appendix B. The results, highlighted in figure B1, show that 3D simulations with a 0.5 mm grid size predicted higher temperature rises compared to 2D simulations. This is attributed to the extended representation of the transducer in the third dimension, which increases the number of grid points and subsequently results in higher temperature predictions. However, 2D simulations with a coarser 0.5 mm grid size effectively captured thermal trends comparable to those observed with a finer 0.05 mm grid size. This finding underscores that, in some cases, coarser grid sizes can be utilized to significantly accelerate simulations without sacrificing accuracy in modeling thermal effects.

### 3.3. FOM analysis with microCT skull model

**Figure 9.**
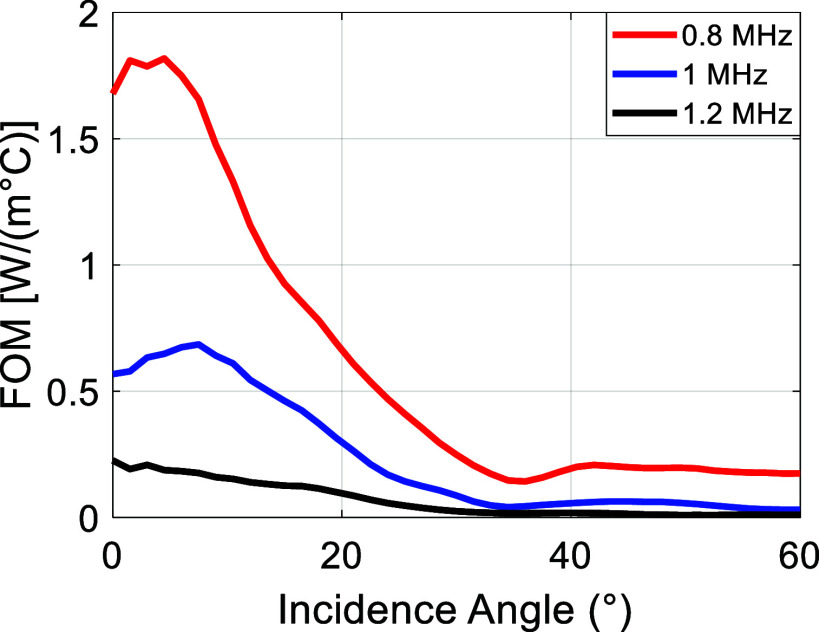
FOM as a function of incidence angle for three different frequencies in shear wave dominant absorption in microCT model: 0.8 MHz (red), 1 MHz (blue), and 1.2 MHz (black).

Figure 9 presents the FOM as a function of incidence angle for three different frequencies: 0.8 MHz, 1 MHz, and 1.2 MHz. The FOM values at 0.8 MHz (red) are consistently higher across all incidence angles compared to 1 MHz (blue) and 1.2 MHz (black), indicating that lower frequencies are more favorable for transcranial ultrasound therapy due to better transmission efficiency and reduced temperature rise. The FOM at 0.8 MHz reaches its maximum value at an incidence angle of around 3°, reduces to half of the peak value around 15° and is significantly low after the longitudinal critical angle (∼30°). This trend suggests that near-normal incidence is the optimal condition for maximizing energy transfer while minimizing thermal effects. For the higher frequencies of 1 MHz and 1.2 MHz, the FOM values are lower across all incidence angles, indicating the effect of increased absorption that reduces transmission efficiency and increases heating within the skull.

**Figure B2.**
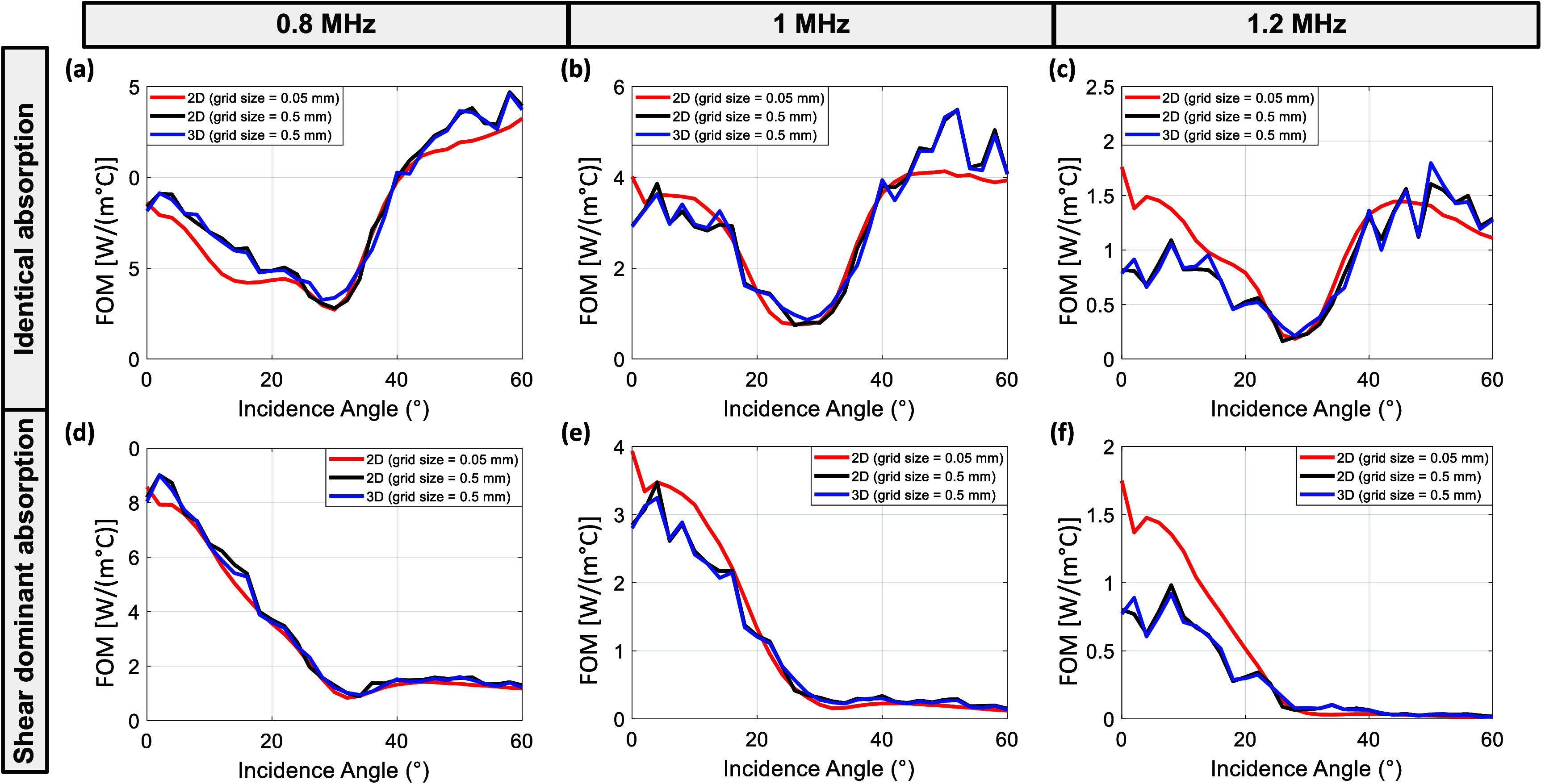
Comparison of the FOM as a function of incidence angle for different simulation configurations. The top row (a)–(c) represents the case with identical absorption for longitudinal and shear waves, while the bottom row (d)–(f) corresponds to shear-dominant absorption. Each column presents results for different frequencies: (a), (d) 0.8 MHz, (b), (e) 1 MHz, and (c), (f) 1.2 MHz. The results are shown for 2D simulations with a 0.05 mm grid size (red), 2D simulations with a 0.5 mm grid size (black), and 3D simulations with a 0.5 mm grid size (blue). The similarity in trends between 2D and 3D simulations suggests that coarser grid sizes can effectively approximate the FOM variations while reducing computational complexity.

**Figure B3.**
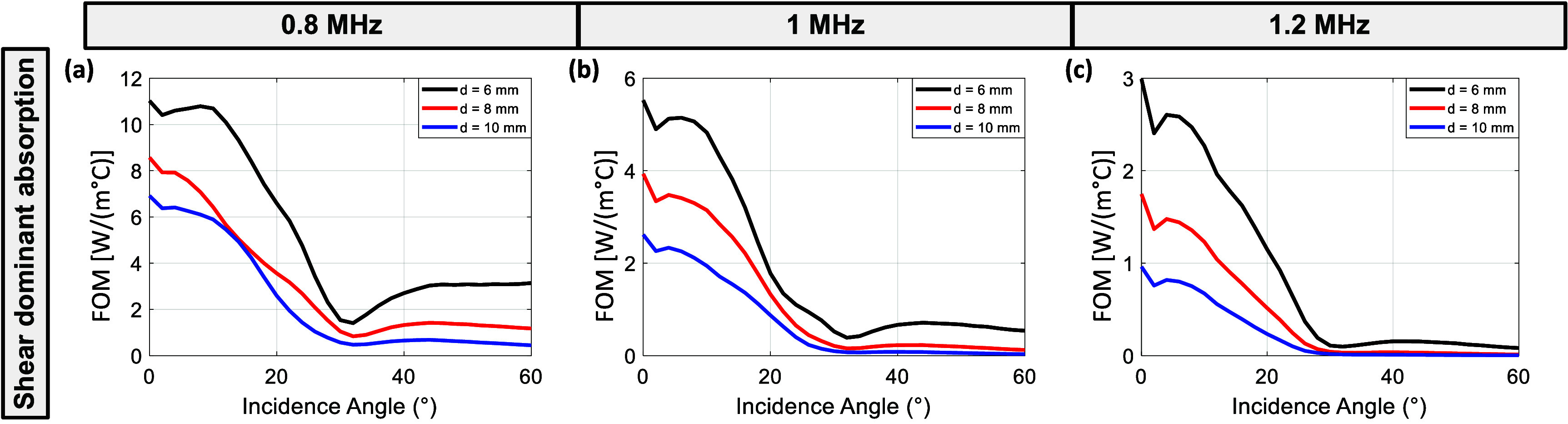
FOM as a function of incidence angle for parallel solid plate skull models of different thicknesses (6 mm, 8 mm, and 10 mm) at ultrasound frequencies of 0.8 MHz (a), 1 MHz (b), and 1.2 MHz (c), showing results for shear-dominant absorption coefficients.

The impact of 2D and 3D simulations and the uniform solid plate approximation on the FOM for various skull thickness values are explored in appendix B. The results demonstrate that with an appropriately fine grid size, the FOM values from 2D and 3D simulations converge (figure B2). Additionally, the overall FOM variation obtained using the solid plate approximation closely mirrors the trends observed in microCT-based FOM analyses. This indicates that, with accurate material properties, the solid plate approach provides a simplified yet effective framework for analyzing FOM (figure B3).

Lastly, in appendix C, figure 1001, we present the FOM results computed using the absorption constants reported by Pinton *et al* (2012). Despite the lower absorption values compared to our main analysis, the overall trend of decreasing FOM with increasing incidence angle remained consistent with the results shown in figure 9. Furthermore, the overall temperature rise distribution also remains similar albeit with smaller magnitudes. This consistency supports the reliability of the FOM trend analysis across different absorption parameter sets, particularly given the approximately 0.5 ratio between longitudinal and shear wave absorption coefficients.

## 4. Discussion

The findings presented in this study highlight the complexity of ultrasound transmission and its associated thermal effects through skull layers, emphasizing the need for an integrated approach that considers both acoustic and thermal phenomena for tFUS applications. A primary contribution of this work is the introduction of FOM which combines ultrasound transmission efficiency with the corresponding thermal impact in the skull. This metric provides a framework for evaluating the performance and safety of tFUS systems and can serve as a guideline for optimizing system parameters such as frequency and incidence angle.

The results demonstrate that while higher resolution simulations (e.g. 0.05 mm grid size) provide more detailed representations of wave propagation and thermal distributions, coarser 2D models with a 0.5 mm grid size can effectively capture the overall transmission behavior when compared to 3D simulations. This was evident in figure 4, where the alignment of transmission coefficients between 2D and 3D models suggests that 2D simulations can serve as a valuable approximation tool when computational resources are limited. Additionally, previous studies have proposed strategies to reduce computational burden, such as segmenting the transcranial simulation domain into specific subregions, thereby limiting the elastic simulations to a smaller area and significantly accelerating the simulation process (Rosnitskiy *et al* 2019). However, higher resolutions are still required for accurately modeling microstructural interactions and wave mode conversions within the skull. The criteria of temporal and spatial sampling has been previously studied in detail to reduce inaccuracies in transcranial ultrasound simulations (Robertson *et al* 2017).

An essential aspect that needs to be considered for large incidence angles is the preservation of the beam profile after transmission through the skull. The 2D scans of the field are shown in figure 5 indicate that although the transmitted beam retains its Gaussian beam profile at 22°, it becomes diffuse at angles around 32°, making it difficult to focus the beams even using transducer arrays (Yousefi *et al* 2009, Bancel *et al* 2021). Variations in skull bone geometries would also introduce further uncertainty to the beam pattern predictions inside the skull at high incidence angles. Consequently, FOM estimations at incidence angles above ∼20° may be overly optimistic, and transducers with larger incidence angles should be considered for inactivation.

Figure 6 highlights the critical role of absorption properties in the skull, as simulations without absorption consistently overestimated the transmission coefficients compared to both experimental measurements and simulations that included absorption. These results contradict previous literature findings, which suggest that only a small portion of the attenuation is due to absorption, with the majority attributed to reflection, scattering, and mode conversion (Pinton *et al* 2012). In contrast, our findings indicate that a significant proportion of the attenuation arises from absorption. Furthermore, while the ratio between longitudinal and shear wave absorption used in our shear wave-dominant model is consistent with other studies (approximately 0.5) (White *et al* 2006, Pinton *et al* 2012, Treeby and Cox 2014, Jing *et al* 2023), the absolute absorption values (8.83 dB cm MHz^−2^ and 19.5 dB cm MHz^−2^) in our simulations are considerably higher than those reported in some other studies (2.7 dB cm MHz^−2^ and 5.4 dB cm MHz^−2^) (White *et al* 2006, Jing *et al* 2023). Although these higher absorption constants yielded results that were more comparable to the experimental measurements in figure 6, it should be noted that they required smaller CFL numbers in elastic simulations to maintain stability.

Thermal simulations using the microCT-based model provided insights into the spatial distribution of temperature rise in the skull. As shown in figure 7, the thermal impact of shear waves becomes more prominent at oblique incidence angles, leading to increased temperature rise in the skull surface. This is consistent with the findings from earlier studies (Treeby and Saratoon 2015, McDannold *et al* 2019), which suggested that mode conversion to shear waves at higher angles results in significant heating within the skull, especially close to the water/skull interface. As observed in figure 8, the contribution of longitudinal wave heating diminishes at higher incidence angles, whereas shear wave heating becomes more significant. The total temperature rise, therefore, reaches its peak at angles where shear waves dominate, reinforcing the need to account for both wave types in the evaluation of thermal safety for transcranial therapies.

**Figure 10.**
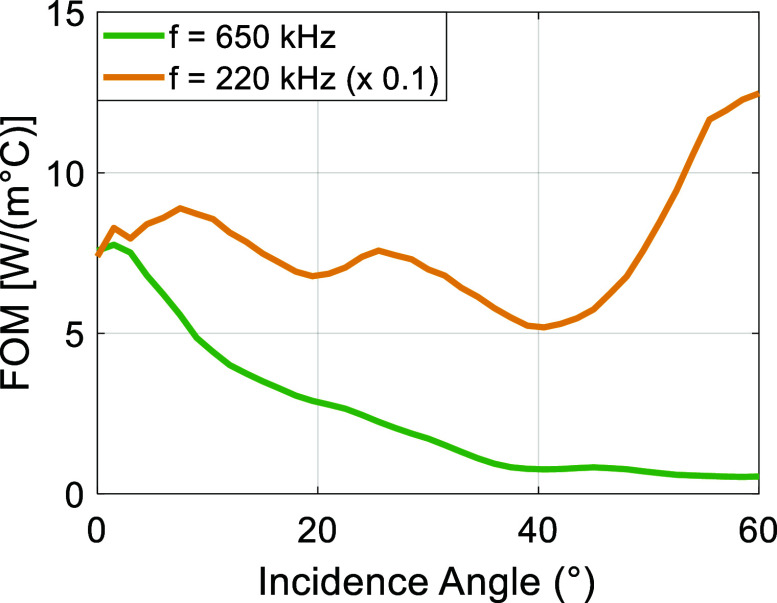
FOM as a function of incidence angle for two clinically relevant frequencies: 650 kHz (green), commonly used in thermal ablation studies, and 220 kHz (orange), often used in BBB opening procedures. 220 kHz FOM curve is scaled by a factor of 0.1 for visual comparison.

The FOM analysis further investigates the influence of frequency and incidence angle on the efficacy and safety of ultrasound transmission through the skull. The results, presented in figure 9, indicate that lower frequencies (e.g. 0.8 MHz) and close to normal incidence result in higher FOM values, making them preferable for transcranial applications. Given the experimentally verified ultrasound parameters, one can extend the FOM simulations to even lower frequencies relevant to clinically approved systems, specifically, 650 kHz for thermal ablation (Martinez-Fernandez *et al* 2020) and 220 kHz for BBB opening applications (Mainprize *et al* 2019). As shown in figure 10, the FOM values 650 kHz follow similar trends as 800 kHz in terms of incidence angle dependence, indicating optimization of incidence angle is important for thermal focused ultrasound treatment. In contrast, at 220 kHz, FOM is significantly higher, ∼10× as compared to 650 kHz for normal incidence (note the 1/10 scaling of FOM for 220 kHz in the graph). It also has much weaker dependence on incidence angle. This is expected due to significantly lower temperature rise at low frequencies. Low frequencies can be advantageous as provide the best compromise between transmission efficiency and temperature rise, but this would limit the focal spot size. Higher frequencies, on the other hand, increase the thermal impact and reduce ultrasound transmission due to higher absorption rates, leading to a decreased FOM and indicating a trade-off between transmission efficiency and thermal safety. This trend suggests that frequency selection should be made with consideration of both transmission and heating effects.

A single flat transducer is used as the example system in our simulations. Assuming linearity of the acoustics and that the incident field can be decomposed into plane waves with different incident angles, this may be a reasonable representation of existing tFUS arrays (Ghanouni *et al* 2015, McDannold *et al* 2019) where multiple unfocused transducers are used to focus the ultrasound beam in the brain. However, the dual mode conversion operation needs to be extended for more complex transducer systems such as concave transducer (Rosnitskiy *et al* 2019, Pouliopoulos *et al* 2020).

Several limitations of this study should be acknowledged. The simulations were primarily conducted in 2D due to computational constraints. While this approach provided significant insights and comparable results between 2D and 3D simulations, future studies should incorporate simulations in a full-scale 3D human skull model to gain a more comprehensive understanding of ultrasound propagation and heating effects. Although this could theoretically be implemented in the current study, a computational system upgrade is required to accommodate the substantial memory demands of the elastic version of *k*-Wave, which requires over 100 GB of RAM. Alternatively, a feasible approach might involve modeling two separate rays within the skull, one longitudinal and one transverse, that merge into a single longitudinal ray within the brain, as described in previous studies (Clement *et al* 2004, Pichardo and Hynynen 2007).

It is also important to note that the skull sample in this study were immersed in water during the experiments. In clinical treatments, however, cold water is circulated, and ultrasound beams pass through multiple tissue layers, including the skin, skull, and various soft tissues such as dura, white matter, grey matter, and cerebrospinal fluid. Despite these differences, we believe they do not significantly impact our findings, as all experimental parameters, except for incidence angle and frequency, were kept constant. Additionally, this study was conducted using a single skull sample to support FOM calculations, which may affect the generalizability of the results. Future work will focus on expanding the sample size to improve statistical significance and further validate the findings.

In addition, further refinement and validation of the reflection, scattering, and absorption modeling remain important directions for future work. Although the current study used literature-based acoustic parameters and microCT-informed geometries, we acknowledge that a more accurate representation of skull heterogeneity may be achieved by incorporating Hounsfield unit-based transformations or estimating acoustic properties from local bone volume fractions (Aubry *et al* 2003, Webb *et al* 2018). Experimental validation of reflected and scattered ultrasound fields, using techniques such as hydrophone measurements with minimal disturbance of the incoming field or Schlieren imaging (Furuhata and Saito 2013), would also strengthen the model’s predictive capability. Likewise, direct measurements of temperature rise, such as with embedded thermocouples or MR thermometry (McDannold *et al* 2004, 2019, 2020), would provide essential confirmation of the simulated heating predictions. While these steps are beyond the scope of the current study, they represent critical next phases in developing a validated simulation framework for safe and effective tFUS. The primary goal of this work was to introduce the FOM as a conceptual tool; future studies will further build upon this framework with refined material modeling and experimental validation.

## 5. Conclusion

This study provides an investigation into ultrasound transmission and its associated thermal effects through skull layers, incorporating both numerical simulations and experimental validation. Our results demonstrate that the combination of longitudinal and shear wave absorptions within the skull plays a significant role in determining ultrasound transmission efficiency and the resulting temperature rise. The findings emphasize the importance of accounting for both wave types when evaluating tFUS systems. The proposed FOM metric, which combines acoustic transmission efficiency with maximum temperature rise, offers a framework for assessing and optimizing the performance and safety of tFUS therapies. Furthermore, the study illustrates that while high-resolution 3D simulations are ideal for capturing complex wave interactions within the skull, appropriately scaled 2D models can offer valuable insights and serve as efficient approximation tools when computational resources are limited. The integration of experimental validation with microCT-derived skull models further reinforces the reliability and accuracy of our simulations. Future research will focus on incorporating more sophisticated skull models and a broader range of samples to improve the predictive capabilities of these models. By considering these factors, the findings from this study can serve as a foundation for optimizing tFUS systems and developing safer and more effective therapeutic interventions.

## Figures and Tables

**Figure A1.**
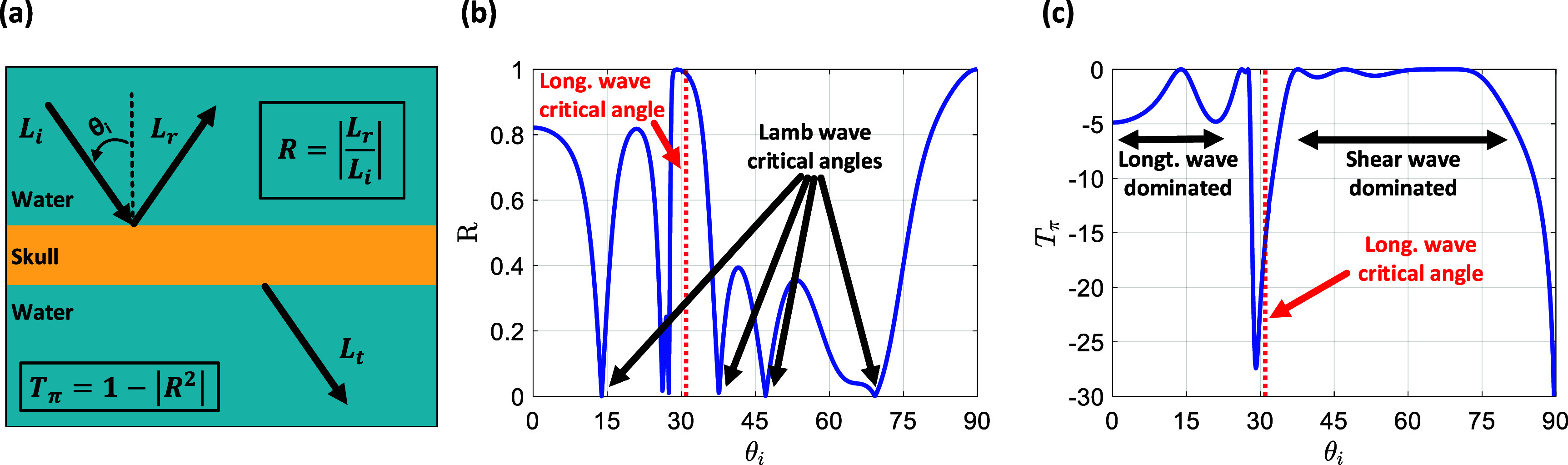
(a) A simplified scheme of ultrasound transmission through immersed skull layer, (b) the magnitude of the plane wave pressure reflection coefficient $\left( R \right)$, and (c) the ultrasound power transmission coefficient $({T_\pi })$ derived from the reflection coefficient as a function of incidence angle $({\theta _{\text{i}}})$.

**Figure C1.**
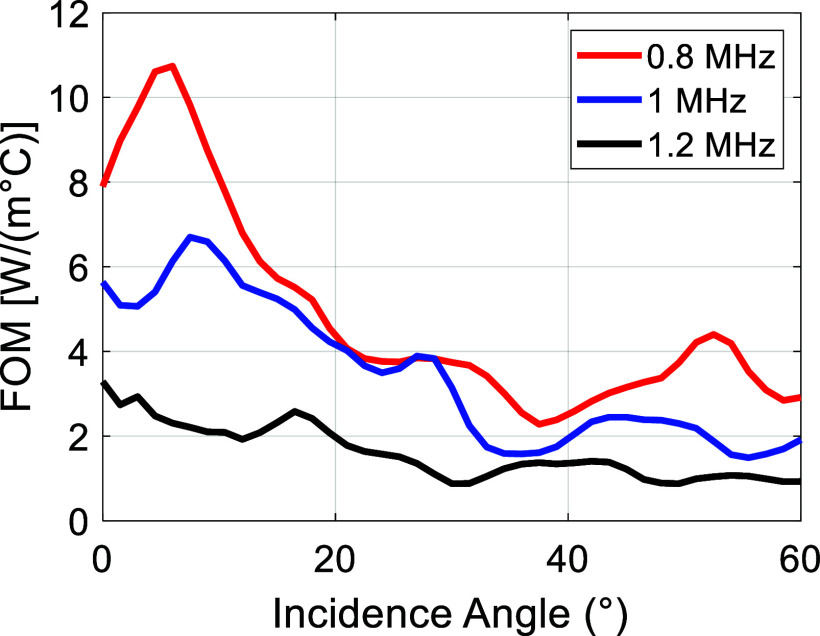
FOM as a function of incidence angle using microCT model simulations with absorption coefficients from Pinton *et al* (2012).

## Data Availability

All data that support the findings of this study are included within the article (and any supplementary information files).

## Appendix A A simplified scheme of ultrasound transmission through immersed skull layer

The fundamental principles of these approaches are illustrated using a parallel solid plate model of the skull, as shown in figure A1, with the properties listed in table 1. The model assumes a generic case with a 6 mm skull thickness and an 800 kHz transmit wave frequency. Figure A1(a) depicts the ultrasound transmission through the skull in an immersed scheme. The magnitude of the plane wave pressure reflection coefficient and the ultrasound power transmission coefficient in dB are presented in figure A1(b) and figure A1(c), respectively, as functions of the incidence angle.

Although this calculation assumes an infinitely wide incident beam with a very narrow angular span and a perfectly uniform, lossless skull layer, it captures the essential Lamb wave-related features for ultrasound transmission through the skull. Key features of this model include transmission primarily governed by the longitudinal wave properties and skull thickness near the normal incidence angle, which is where most tFUS systems operate. Starting around 15°, various Lamb wave modes are excited, leading to dips in the reflection coefficient. As these waves propagate laterally in the skull, they radiate energy into the brain, creating peaks in the power transmission plot at critical angles, indicative of nearly perfect transmission.

Additionally, an angular span around the longitudinal critical angle exhibits high reflection and significantly reduced transmission, which should be avoided. Beyond this angle, Lamb wave modes are predominantly shear wave components, resulting in a broad range (approximately 35°–75°) of efficient transmission. This broad angular range of efficient transmission forms the basis for the dual-mode conversion approach (Kang *et al* 2022).

## Appendix B Additional assessment of grid resolution, dimensionality, and skull thickness on heating and FOM in parallel solid plate skull models

In figure B1, with a shear-dominant absorption model, temperature rise increases significantly with incidence angle, following a similar trend across grid sizes and dimensions. However, 3D simulations consistently show higher temperature rises. The increased number of grid points representing the 12.7 mm diameter transducer element in 3D simulations amplifies the incoming pressure amplitude on the skull surface, resulting in a higher peak temperature rise.

The FOM was calculated for various scenarios to compare 2D and 3D simulations. Figure B2 presents the FOM as a function of incidence angle for different models using 2D simulations with 0.05 mm and 0.5 mm grid sizes, as well as 3D simulations with a 0.5 mm grid size. Simulations were conducted at frequencies of 0.8 MHz, 1 MHz, and 1.2 MHz with shear-dominant absorption for an 8 mm skull thickness. The 3D FOM values were scaled using integrated power on the skull surface, normalized by skull temperature from the 2D simulations, to facilitate direct comparison.

The FOM results reveal that while overall trends are consistent across grid sizes and dimensions, simulations at 1.2 MHz exhibit more significant discrepancies between 0.05 mm and 0.5 mm grid sizes. This difference likely stems from the reduced PPW, which leads to higher staircase errors, decreasing spatial resolution and impairing representation of the transducer’s interaction with the skull. Nonetheless, the close alignment of 2D and 3D results at different angles suggests that appropriately scaled 2D simulations can provide valuable insights for optimizing transcranial ultrasound therapy parameters.

The FOM was calculated across various skull thicknesses in parallel solid plate models to evaluate the impact of skull thickness on ultrasound transmission and heating. At 0.8 MHz (figure B3(a)), the FOM decreases sharply with increasing incidence angle, with the 6 mm skull consistently exhibiting the highest values due to its higher transmission efficiency compared to thicker skulls. At 1 MHz (figure B3(b)), a similar trend is observed, with the FOM declining steeply as the incidence angle increases. The 6 mm skull maintains the highest FOM, with minimum values occurring around 20°–30°, followed by stabilization at higher angles. At 1.2 MHz (figure B3(c)), the FOM values are generally lower across all thicknesses compared to lower frequencies. Nonetheless, the 6 mm skull continues to exhibit the highest FOM, although a noticeable decline is evident at higher incidence angles. These findings emphasize the significant influence of both skull thickness and sonication frequency on FOM, with thinner skull models and lower frequencies yielding higher transmission efficiency. Furthermore, for the same frequency, the thermal impact appears to be relatively unaffected by variations in skull thickness, as the peak temperature rise consistently occurs near the surface of the skull, as shown in figure 7.

## Appendix C FOM analysis with microCT skull model using lower absorption coefficients

To evaluate the sensitivity of FOM to different absorption parameter sets, we repeated the microCT-based simulations using the absorption constants reported by Pinton *et al* (2012), which are widely adopted in transcranial ultrasound simulation studies (Jing and Lindsey 2021, Jing *et al* 2023, Manuel *et al* 2023). Specifically, longitudinal and shear wave absorption coefficients were set to 2.7 dB (MHz^2^ cm)^−1^ and 5.4 dB (MHz^2^ cm)^−1^, respectively, substantially lower than the values used in our main analysis (8.83 and 19.5 dB (MHz^2^ cm)^−1^).

As expected, this led to higher FOM values due to increased ultrasonic transmission and reduced thermal deposition. Nevertheless, the overall trend of decreasing FOM with increasing incidence angle remained consistent with the results presented in figure 9. This consistency highlights the behavior of the FOM framework in capturing relative performance trends across varying incidence angles, particularly when the ratio between longitudinal and shear wave absorption coefficients (∼0.5) is preserved.
